# Coresidence Increases Risk of Testing Positive for COVID-19 Among Older Brazilians

**DOI:** 10.1093/geroni/igab046.3475

**Published:** 2021-12-17

**Authors:** Flavia Andrade, Nekehia Quashie, Luisa Schwartzman

**Affiliations:** 1 University of Illinois--Urbana-Champaign, Urbana, Illinois, United States; 2 Technical University of Dortmund, Dortmund, Nordrhein-Westfalen, Germany; 3 University of Toronto, Mississauga, Ontario, Canada

## Abstract

Brazil is among the countries hit hardest by COVID-19, and older adults are among the vulnerable groups. Intergenerational coresidence and interdependence among family members, both prevalent in Brazil, likely increase social and physical contact. Using nationally representative data from the COVID-19 module of the Brazilian National Household Sample Survey, collected from July to November of 2020, we examined the association between living arrangements and exposure to and testing for COVID-19 among 63,816 Brazilians 60+. Our multivariate analyses utilize multilevel mixed-effects Poisson regression to examine the association between living arrangements and the COVID-19 outcome measures. Results show that those living alone were more likely to report having symptoms and having had a test for COVID-19. However, older adults in multigenerational (PR=1.532, 95% CI 1.15, 2.04, p<0.001) and skipped generation households (PR=1.607, 95% CI 1.04, 2.48, p<0.001) were more likely than solo-dwellers to test positive for COVID-19. Those with symptoms were more likely to test, regardless of their living arrangement. Among older adults without symptoms, those living alone had a higher probability of testing than those living in multigenerational or skipped-generation households. Overall, our findings suggest that coresidence with younger family members is a risk factor for older adults’ health due to the higher COVID-19 positivity. As younger Brazilians are increasingly vulnerable to COVID-19 and experiencing severe outcomes, policy makers need to be more attentive to the health needs of households that comprise older and younger cohorts, which are also more prevalent in poor and marginalized segments of the population.

